# Elevation in the counts of IL-35-producing B cells infiltrating into lung tissue in mycobacterial infection is associated with the downregulation of Th1/Th17 and upregulation of Foxp3^+^Treg

**DOI:** 10.1038/s41598-020-69984-y

**Published:** 2020-08-06

**Authors:** Chen Chen, Huan Xu, Ying Peng, Hong Luo, Gui-Xian Huang, Xian-Jin Wu, You-Chao Dai, Hou-Long Luo, Jun-Ai Zhang, Bi-Ying Zheng, Xiang-Ning Zhang, Zheng W. Chen, Jun-Fa Xu

**Affiliations:** 1 Department of Clinical Immunology, Institute of Clinical Laboratory Medicine, Guangdong Provincial Key Laboratory of Medical Molecular Diagnostics, Guangdong Medical University, No. 1 Xincheng Road, Dongguan, 523808 China; 2 Molecular Diagnostic Center, The Sixth Affiliated Hospital of Guangzhou Medical University, Qingyuan People’s Hospital, Qingyuan, 511518 China; 3 Department of Clinical Laboratory, Huizhou Municipal Central Hospital, No. 41 North Eling Road, Huizhou, 516001 China; 4 Department of Pathophysiology, Basic Medical School, Guangdong Medical University, No. 1 Xincheng Road, Dongguan, 523808 China; 5 Department of Microbiology and Immunology, Center for Primate Biomedical Research, University of Illinois College of Medicine, Chicago, IL USA

**Keywords:** Infectious diseases, Microbiology

## Abstract

IL-35 is an anti-inflammatory cytokine and is thought to be produced by regulatory T (Treg) cells. A previous study found that IL-35 was upregulated in the serum of patients with active tuberculosis (ATB), and IL-35-producing B cells infiltrated to tuberculous granuloma of patients with ATB. Purified B cells from such patients generated more IL-35 after stimulation by antigens of *Mycobacterium tuberculosis* and secreted more IL-10. However, the function and the underlying mechanisms of IL-35-producing B cells in TB progression have not been investigated. The present study found that the expression of mRNA of IL-35 subsets *Ebi3* and *p35* was elevated in mononuclear cells from peripheral blood, spleen, bone marrow, and lung tissue in a mouse model infected with *Mycobacterium bovis* BCG, as tested by real-time polymerase chain reaction. Accordingly, the flow cytometry analysis showed that the counts of a subset of IL-35^+^ B cells were elevated in the circulating blood and in the spleen, bone marrow, and lung tissue in BCG-infected mice, whereas anti-TB therapy reduced IL-35-producing B cells. Interestingly, BCG infection could drive the infiltration of IL-35-producing B cells into the lung tissue, and the elevated counts of IL-35-producing B cells positively correlated with the bacterial load in the lungs. Importantly, the injection of exogenous IL-35 stimulated the elevation in the counts of IL-35-producing B cells and was associated with the downregulation of Th1/Th17 and upregulation of Foxp3^+^Treg.The study showed that a subset of IL-35-producing B cells might take part in the downregulation of immune response in mycobacterial infection.

## Introduction

Tuberculosis (TB), caused by *Mycobacterium tuberculosis* (*Mtb*), commonly known as tubercle bacillus, is a major health problem worldwide, with approximately one third of the global population infected and 10 million new cases reported officially to the World Health Organization in 2017^1,2^. The susceptibility to the onset and progression of TB involves not only the damage of infected tissues but also the deregulation of host immune response to the pathogen^3^. Innate immune cells mediating bactericidal effects and subsequent activation of T cell—mediated adaptive immunity play an important role in the clearance of tubercle bacillus^4^. *Mtb*-induced T cell immune response involves several conventional (αβ T cells) and unconventional (γδ T cells and CD1-restricted cells) T cell subsets^5^. *Mtb*-specific αβ T cells, such as Th1, Th17, and cytolytic CD8^+^ T cells, play an important role in protective immunity against TB^6,7^, but regulatory T cells are always downregulated in anti-TB immunity^8^. The role of B cells in anti-tuberculosis immunity, however, has long been ignored. Studies suggested that B cells could present antigen for effective priming of T cells and promote humoral immunity in the control of *Mtb* infection^9–11^. Recently, Evidences showed that B cells also played a critical role as regulators to promote anti-TB immunity by producing cytokines^9–11^. On the contrary, some recent studies suggested that B cells could also accelerate the progression of infectious diseases^12–16^. In the recent years, a novel subset of B cells, which exert immune-regulatory effects by producing interleukine (IL)-10, transforming growth factor-β (TGF-β), and IL-35, have been identified and classified as regulatory B cells (Bregs). IL-10-producing B cells was first found in chronic intestinal inflammation^17^. They have also been reported in autoimmune diseases^18^^,^ allergic diseases^19^^,^ graft-versus-host disease^20^^,^ and cancer^21^. In *M. tuberculosis* infections, a subset of CD19^+^CD1d^+^CD5^+^ Bregs was found associated with active tuberculosis (TB)^22^. Subsequent study^23^ revealed that successful anti-TB treatment in human TB induced an increased IL-22 response by reducing the frequencies of CD19^+^CD5^+^CD1d^+^Bregs. Notablely, it was found that patients with cavitary TB had significantly higher frequencies of CD19^+^CD1d^+^CD5^+^ B cells. In a mouse model, B-cell subpopulation expressing IL-10 downregulated proinflammatory cytokine expression in the spleen, increasing the survival of CD4^+^ TEM cells and CD8^+^ TEM/CD127^+^ cells^24^. More recently, a study reported that *M. tuberculosis* mannose-capped lipoarabinomannan induces IL-10-producing B cells and hinders CD4^+^Th1 immunity^25^. These findings suggested that IL-10-producing Bregs impaired protective immunity and increased disease severity. A novel subset of Bregs producing an anti-inflammatory cytokine, IL-35, was reported during *Salmonella* infections and experimental autoimmune encephalomyelitis (EAE). These cells play a key role in immunosuppression^26,27^.

IL-35 is composed of the p35 subunit of IL-12 and the subunit of IL-27 of Epstein-Barr Virus (EBV)-induced gene 3 (EBI3) and appears to be produced exclusively by Treg cells, DC cells, macrophages, human placental trophoblasts, and a variety of tumor tissues^28–30^. IL-35 appears to suppress T cell proliferation and differentiation, and induces Treg cells polarization^28,31^. Similar to IL-10 and TGF-β, IL-35 can also ameliorate autoimmune diseases and promote the development of infectious diseases. A previous study showed that patients with active TB exhibited increases in serum IL-35 levels and the mRNA expression of both subunits of IL-35 (*p35* and *EBI3*) in white blood cells and peripheral blood mononuclear cells. *Mtb* infection was associated with the expression of p35 or EBI3 protein in CD4^+^ but not in CD8^+^ T cells. Most p35^+^CD4^+^ T cells and EBI3^+^CD4^+^ T cells expressed the Treg-associated marker CD25^32^. More recently, it was reported that IL-35-producing B cells infiltrated into the tuberculous granuloma of patients with ATB, and the mRNA expression of both subunits of IL-35 (*p35* and *Ebi3*) in purified B cells from patients with ATB was elevated. Furthermore, purified B cells from patients with ATB produced more IL-35 after stimulation by *Mtb* antigen, and the IL-35-producing B cells expressed the high level of IL-10^33^. However, the function and underlying mechanisms of IL-35-producing B cells in TB progression have not been investigated.

In the present study, a mouse model infected with *Mycobacterium bovis* bacillus was used to further validate previous findings in patients with TB that *Mtb* infection could induce the increase in IL-35-producing B cells and their infiltration into TB lesion tissue. In addition, this study examined the effects of IL-35-producing B cells on the bactericidal activity in the mouse model. It also explored the correlation between IL-35-producing B cells and the effector/regulatory T cells in mycobacterial infection.

## Materials and methods

### Antibodies and reagents

Bacterial culture media used in the study were 7H9 Roche solid medium (Baso, Zhuhai, China), 7H9 liquid medium and Middlebrook 7H11 agar plates supplemented with 10% oleicacid–albumin–dextrose–catalase (OADC) (HiMedia Laboratories). A mouse tissue mononuclear cell separation kit was purchased from TBDsciences (Tianjing, China). Collagenase (Sigma, USA), trypsin inhibitor (Soleil, China), Roswell Park Memorial Institute (RPMI) 1640, and Fetal Bovine Serum (FBS, Gibco, USA) were used in the study. Immunohistochemistry (IHC) and immunofluorescence (IF) staining reagents included hematoxylin–eosin dye (BeyotimeBiotechnology, Beijing, China), acid-fast dyeing kit (Baso, Wuhan, China), and 4′,6-diamidino-2-phenylindole (DAPI) staining solution (Beyotime Biotechnology, Beijing, China). The reagents were used also as follows: goat serum (Gibco, USA), anti-fluorescence quenching sealant (Beyotime Biotechnology, Beijing, China), mouse B220/CD45R antibody (R&D, USA), interleukine (IL)-27/IL-35 EBI3 subunit antibody (Novus Biologicals, USA), anti-mouse p35 FITC or p35 APC (R&D), anti-rat IgG(H + L), (Alexa Fluor 647 Conjugate), and anti-rabbit IgG (H + L), F(ab)_2_ fragment (Alexa Fluor 594 Conjugate) (Cell Signaling Technology, USA). Quantitative real-time polymerase chain reaction (qRT-PCR) analysis involved the use of TRIzol reagent (Ambion, Shanghai, China), reverse transcription kit (TaKaRa, RR047A, Dalian, China), RT-PCR system (Roche, LightCycler96, Shanghai, China), and PCR parameters (TRANSGene, Beijing, China). Flow cytometry (FCM) and intracellular cytokine staining antibodies, anti-mouse-CD19-PE or CD19-FITC, anti-mouse-B220-percp,anti-mouse-P35-FITC or P35 APC, and anti-mouse-EBI3-APC, were from BD Company (USA). Anti-mouse-IL-10-PE (JES5-16E3, eBioscience) was used for the detection of IL-10. APC-anti-mouse CD4 antibody (eBioscience), PE-cy7-anti-mouse IFN-γ antibody (Thermo Fisher Scientific, USA), and PE-anti-mouse IL-17 antibody (eBioscience) were used for detecting Th1 or Th17. PE-cy7 anti-mouse CD3 antibody and FITC anti-mouse CD4 antibody (Biolegend, Beijing, China), APC-anti-mouse CD25 antibody, and PE-cy5-anti-mouse-Foxp3 antibody (eBioscience) were used for detecting Foxp3 + Treg. Recombinant mouse IL-35—human Fc chimeric protein was purchased from Chimerigen Laboratories (CA, USA)^34^. Rifampicin (RIF) and isoniazid (RIF) were from Hongqi Pharmaceutical Co., Ltd. in Shenyang and Minsheng Pharmaceutical Co., Ltd. in Hangzhou, China, respectively.

### Bacterial strains and culture conditions

*Mycobacterium bovis* BCG (from the laboratory of Zheng W. Chen at the University of Illinois at Chicago) was grown in liquid Middlebrook: broth 7H9 medium (HiMedia Laboratories) supplemented with 0.2% (*v*/*v*) glycerol (Beijing Modern Eastern Fine Chemicals, Beijing, China), 0.05% Tween 80 (*v*/*v*) (Sigma), and 10% ADS [50 g/L bovine serum albumin (BSA), 20 g/L dextrose, and 8.5 g/L NaCl]in a CO_2_ incubator (37 ℃, 5% CO_2_) and cultured with shaking (240 rpm) for 4–6 weeks. Then, the bacilli were harvested, ground thoroughly in sterile 7H9 liquid medium, and diluted appropriately. Each bacterial dilution was inoculated in Roche solid medium (BaSo, Zhuhai, China) and then cultured in a CO_2_ incubator with 5% CO_2_ for 4–6 weeks, and the colonies were counted for live bacteria per millet unit.

### Mouse model and specimen collection

Wild-type C57BL/6J mice were purchased from the Laboratory Animal Center of Southern Medical University, certified by the Laboratory of Animal Quality of Guangdong Province (license No. SCXK (Guangdong) 2016-0041). All female mice, aged 6–8 weeks, were used and randomized for the experiments. The treatment process and experimental procedures were in accordance with the regulations of the experimental animal management. C57BL/6J mice were divided into BCG infection experimental group and saline control group. The experimental group was infected with BCG via the tail vein at a dose of 2 × 10^6^ colony formation unit (CFU)/200 μL, and the control group was injected with 200 μL of saline. The mice were euthanized, and efforts were made to minimize suffering. Blood, spleen, bone marrow, and lung tissues were collected for isolating mononuclear cells at different time points (2, 4, and 8 weeks) after infection for detecting IL-35 subsets. *Ebi3* and *p35* expression was analyzed by real-time RT-PCR, while the protein expression was analyzed by flow cytometry with intracellular cytokine staining at each time point^35^. Lung tissues were also dissected aseptically for histological and pathological analyses and for determining the bactericidal activity via direct bacterial counting. A total of 10 mice were selected from each group. Attention was paid to the matching of age, weight, and physiological status among the groups.

### Exogenous IL-35 or anti-TB drug treatment

The BCG-infected mouse model was prepared as described as earlier. Ten mice were treated with exogenous IL-35. One week after BCG infection, the mouse model was intraperitoneally injected with 0.8 µg IL-35 protein once a week for a total of three injections^34,36^, namely BCG + IL-35 protein group. The mice treated with an equal volume of saline were used as the control group. Another 10 mice were orally administered rifampicin (RIF, 200 mg/L) and isoniazid (INH, 100 mg/L), through daily drinking water, for 3 weeks after 1 week of BCG infection, namely RIF + INH group.

### Mycobacterial colony formation unit count

Lung tissues were harvested aseptically at different time points (2, 4, and 8 weeks) after infection. Under sterile conditions, 0.04 g lung tissue was taken from the left lower lobe of the mice. Then, 500 μL of saline was added to the grinder and diluted with an equal volume of 4% NaOH for 10 min. A tenfold serial dilution was performed for quantitative culturing. Aliquots (100 μL) were coated in triplicate on Middlebrook 7H11 agar plates supplemented with 10% oleicacid–albumin–dextrose–catalase (OADC) enrichment for 4–6 weeks of culture in a CO_2_ incubator with 5% CO_2_ until colonies were large enough to be counted. The mycobacteria viability was quantified by counting CFUs.

### Isolation of peripheral blood mononuclear cells and tissue mononuclear cells

Peripheral blood mononuclear cells (PBMCs) and bone marrow mononuclear cells were prepared as described previously^37,38^. Blood samples or bone marrow irrigating solution was collected in tubes precoated with heparin sodium for anticoagulation. PBMCs or bone marrow mononuclear cells were freshly isolated from blood by standard Ficoll (GE Healthcare) density gradient centrifugation. The cell viability was determined by trypan blue exclusion (> 95% in all experiments).

The lung tissue was aseptically dissected. After washing three times with saline to clean the blood, the tissue was homogenized and incubated at room temperature for 30 min with 5 mL of enzymatic hydrolysate [containing 0.05% (*w*/*v*) collagenase and 0.01% (w/v) trypsin inhibitor in RPMI1640]. Then, the tissues lysate was gently milled on a 200-mesh filter with a grinding rod. A mouse tissue mononuclear cell separation kit for isolated cells (TBDsciences) was used for lung mononuclear cell (LMC) isolation. Splenic cells were isolated from spleens of mice by gradient centrifugation with Ficoll solution (*d* = 1.088). The cell viability was determined by trypan blue exclusion (> 95% in all experiments).

### Histological analysis and IF staining

The lung tissue (about 1 cm^3^) was collected at necropsy and fixed in 10% paraformaldehyde in phosphate-buffered saline (PBS). Randomly selected tissue sections were embedded in paraffin and cut into 5-μm sections on a microtome. The tissue microsections were mounted on glass slides, deparaffinized, and stained with hematoxylin–eosinand carbolfuchsin by the Ziehl–Neelsen method, as reported previously^39^, and then analyzed by a certified pathologist.

For IF staining, the paraffin sections of the lung tissue were dried and dewaxed. The antigen was repaired and blocked with a blocking solution containing 4% BSA and 5% goat serum (Gibco) for 1 h. Then, 50 μL of pre-mixed primary antibodies (mouse B220/CD45R antibody, R&D; IL-27/IL-35 EBI3 subunit antibody, Novusbio; anti-Mouse P35 FITC) was added to the corresponding tissue blocks and incubated overnight at 4 °C. The concentration of the primary antibodies was diluted according to the recommended folds in the manual. On the next day, the slides were equilibrated at room temperature for 1 h. After washing with PBST, the diluted fluorescently labeled secondary antibody was added dropwise to the tissue sections and incubated at room temperature for 1 h in the darkness. The secondary antibodies anti-rat IgG (H + L) (Alexa Fluor 647 Conjugate, Cell Signaling Technology) and anti-rabbit IgG (H + L), F(ab)_2_ Fragment (Alexa Fluor 594 Conjugate, Cell Signaling Technology) were diluted (1:1,000) according to the recommended amount in the instruction manual and rinsed with PBST. Finally, confocal images were captured with a laser scanning confocal microscope LSM800 (Zeiss, Jena, Germany).

### Quantitative real-time polymerase chain reaction analysis

Total RNA was extracted from PBMCs and mononuclear cells from bone marrow, spleen, and lung tissue in the TRIzol reagent according to the procedures recommended by the manufacturer (Ambion). All RNA samples were purified by phenol/chloroform/alcohol extractions and precipitated in 10 μL of DEPC water. Then, 20 μL (10 ng/μL) mRNA was reverse-transcribed to cDNA using a PrimeScript RT Reagent Kit (RR047A, TaKaRa, Dalian, China), and the expression of Ebi3 and p35 genes was analyzed. GADPH was used as an internal control. The primer sequences were as follows: *EBI3*: 5′-CCTTTGTGGCTGAGCGAATC-3′ (forward) and 5′-CACCTGGCGGAAGTGAGA-3′ (reverse); *p35*:5′-AGCGTTCCAACAGCCTCA-3′ (forward) and 5′-GCTGGTTTGGTCCCGTGT-3′ (reverse); and GADPH: 5′-CCTTCCGTGTTCCTACCC-3′ (forward) and 5′-GCCCTCAGATGCCTGCT-3′ (reverse). RT-PCR was performed on a fast RT-PCR system (Roche, LightCycler96), and PCR amplification were as recommended by the manufacturer (TRANSGene). Mouse GADPH, *p35*, and *EBI3* primer sequences were designed by Primer5.0 combined with PubMed design, synthesized by Shanghai Shenggong Bioengineering Co., Ltd. For the relative quantitation of gene expression, the 2^−ΔΔ^CT method was used, with GADPH as the internal control for normalization^34^.

### Flow cytometry analysis and intracellular cytokine staining

Flow cytometry (FCM) analysis and intracellular cytokine staining were performed as previously described. PBMCs, LMCs, and mononuclear cells from the spleen and bone marrow were used for detecting IL-35 or IL-10 expression in B cells, while PBMCs and LMCs were used for detecting IFN-γ, IL-17, and Foxp3 expression in CD4 + T cells, by direct intracellular cytokine staining (ICS) without stimulation in vitro^35,40^. PBMCs, LMCs, and mononuclear cells from the spleen and bone marrow were directly stained with anti-mouse-p35-FITC (R&D) and anti-mouse-EBI3-APC (R&D) for detecting IL-35 or stained with anti-mouse-IL-10-PE (JES5-16E3, eBioscience) for detecting IL-10. APC-Anti-mouse-CD4 antibody (eBioscience) and PE-cy7-anti-mouse-IFN-γ antibody (Thermo Fisher Scientific) or PE-anti-mouse-IL-17 antibody (eBioscience) were used for detecting Th1 or Th17. PE-cy7-anti-mouse-CD3 antibody (Biolegend), FITC-anti-mouse-CD4 antibody (Biolegend), APC-anti-mouse-CD25 antibody (eBioscience), and PE-cy5-anti-mouse Foxp3 antibody (eBioscience) were used for detecting Foxp3^+^ regulatory T cells. ICS was performed as previously described^35^. The cells were washed once with 2% FBS–PBS and stained at room temperature for 25 min with surface marker Abs. For ICS, PBMCs, LMCs, or mononuclear cells of spleen and bone marrow were further washed twice with 2% FBS–PBS, permeabilized with BD FACS permeabilizing solution (BD Biosciences) for 30 min at room temperature, and then stained for another 45 min with cytokine antibodies, followed by two final washes with 2% FBS–PBS buffer and analysis with BD FACSVerse (BD Biosciences) flow cytometry. To ensure specific immune staining in ICS, matched isotype IgG served as negative control for staining cytokines or surface markers.

### Statistical analysis

Flow cytometry data were analyzed by FlowJo software 7.6 (Tree Star Inc., CA, USA). Statistical analysis was carried out using GraphPad Prism 5 software (GraphPad Software Inc., CA, USA). The difference between the two groups was analyzed using the Student *t* test. The data of multiple groups were compared by analysis of variance. The correlation was analyzed using Pearson coefficient A *P* value less than 0.05 was considered statistically significant.

### Ethics statement

This study was performed in strict accordance with the recommendations in the Guide for the Care and Use of Laboratory Animals, and the protocol was approved by the Ethics Committee of Guangdong Medical University (GDMU). The mice were bred under specific pathogen-free conditions in the laboratory animal facility at GDMU. All animal experiments were conducted under isoflurane anesthesia, and all efforts were made to minimize suffering.

## Results

### Mycobacterial infection induced high mRNA expression of IL-35 subsets *Ebi3* and *p35* in PBMCs and immune tissue mononuclear cells

**Figure 1:**
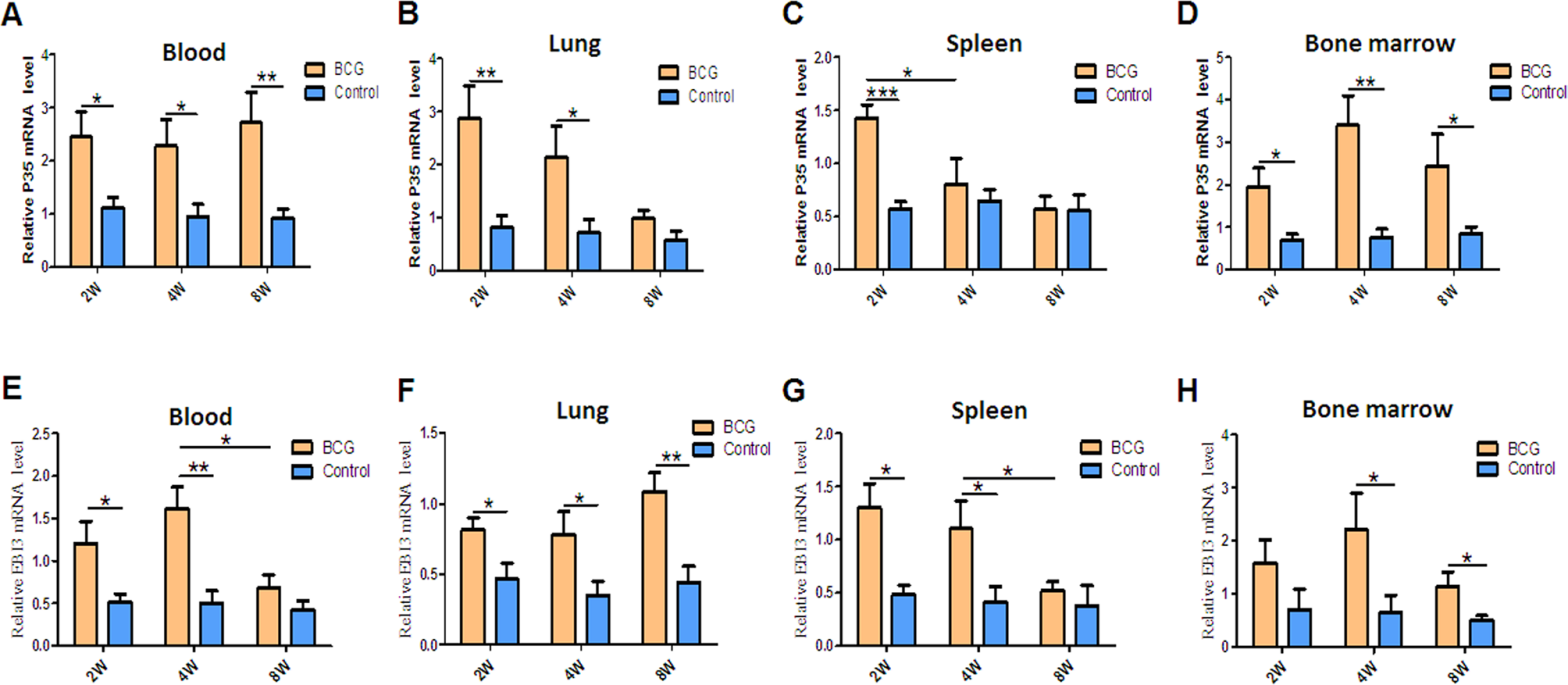
*Mycobacterium bovis* BCG infection induced high expression of mRNA of IL-35 subsets *Ebi3* and *p35* in mononuclear cells in the peripheral blood and in immune tissues in mice. PBMCs and mononuclear cells from lung (LMC), spleen, and bone marrow were extracted from *Mycobacterium bovis* BCG-infected mice (*n* = 6). Then, the cells were assayed for IL-35 subsets *p35* and *Ebi3* mRNA expression using real-time PCR. (**A**–**D**) Relative mRNA expression of IL-35 subset *p35* in the peripheral blood (**A**), lung (**B**), spleen (**C**), and bone marrow (**D**). (**E**–**H**) Relative mRNA expression of IL-35 subset *Ebi3* in the peripheral blood (**E**), lung (**F**), spleen (**G**), and bone marrow (**H**). The *P* value is shown in each column. *P < 0.05; **P < 0.01; ***P < 0.001.

**Figure 2:**
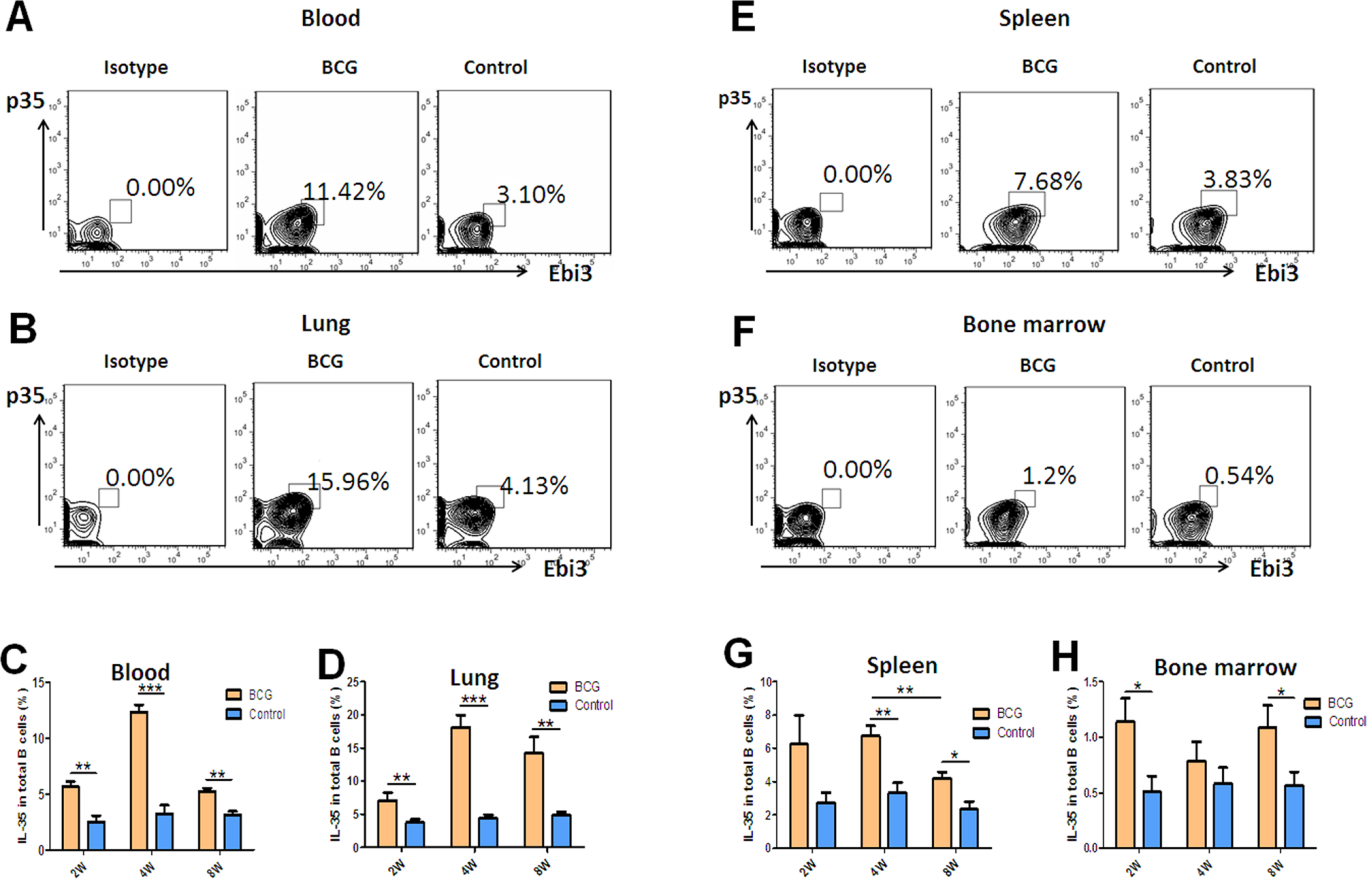
BCG infection induced the elevation in the counts of IL-35-producing B cells in the blood and in each immune tissue. PBMCs and mononuclear cells from the lung, spleen, and bone marrow were extracted from BCG-infected mice (*n* = 6). Then, the cells were assessed for the frequencies of IL-35-producing B cells by flow cytometry. The gating strategy is shown in Figure S2. (**A**, **B**, **E**, **F**) shows the representative histograms for flow cytometry analysis of the expression of IL-35 in B cells in PBMCs (**A**) and B cells in mononuclear cells from lung (**B**), spleen (**E**), and bone marrow (**F**) in BCG-infected mice and controls for 4 weeks. (**C**, **D**, **G**, **H**) shows the graph data showing the mean frequencies of IL-35-producing cells in B cells in PBMCs (**C**) and in mononuclear cells from the lung (**D**), spleen (**G**), and bone marrow (**H**) in BCG-infected mice (*n* = 10) for 2, 4, and 8 weeks and controls (*n* = 6). The *P* value is shown in each column. *P < 0.05; **P < 0.01; ***P < 0.001.

A recent study reported that IL-35 was produced by not only T cells^26,28^ but also B cells^27^. A previous study showed that IL-35 was upregulated in serum in patients with active TB and was related to the activation of Treg cells^32^. IL-35 was produced by B cells, with a strong ability to produce IL-10, in the peripheral blood in patients with active TB and in human tuberculous granuloma^33^. For further understanding the roles of IL-35 played in pulmonary tuberculosis, a mouse model was prepared with *M. bovis* BCG infection (Fig. S1), and mononuclear cells were isolated from peripheral blood, lung, spleen, and bone marrow. Subsequently, the mRNA and protein expression of IL-35 subsets *p35* and *Ebi3* in these cells was determined by real-time PCR and flow cytometry with ICS. Interestingly, the expression of p35 and Ebi3 genes increased in mononuclear cells in the blood and in each tissue 2, 4, and 8 weeks after BCG infection (Fig. 1), especially in the circulating blood and lung tissue (Fig. 1). Notably, flow cytometry with ICS analysis also showed that the frequencies of p35^+^ and Ebi3^+^ B cells were much higher in BCG-infected mice than in control mice 2, 4, and 8 weeks after BCG infection, which was consistent with the mRNA expression (Fig. 2). Intriguingly, the frequencies of p35^+^ and Ebi3^+^ B cells were much higher (about 1.5 times) in the lungs than in the circulating blood, indicating that BCG infection might drive the infiltration of IL-35-producing B cells into the lungs (Fig. 2E, F).

Together, these results suggested that a subset of B cells was distributed in immune organs. BCG infection induced the high expression of IL-35 in B cells in the peripheral blood and lung tissue, and also caused the infiltration of B cells in mice.

### BCG infection-driven infiltration of IL-35-producing B cells into lung tissue

**Figure 3:**
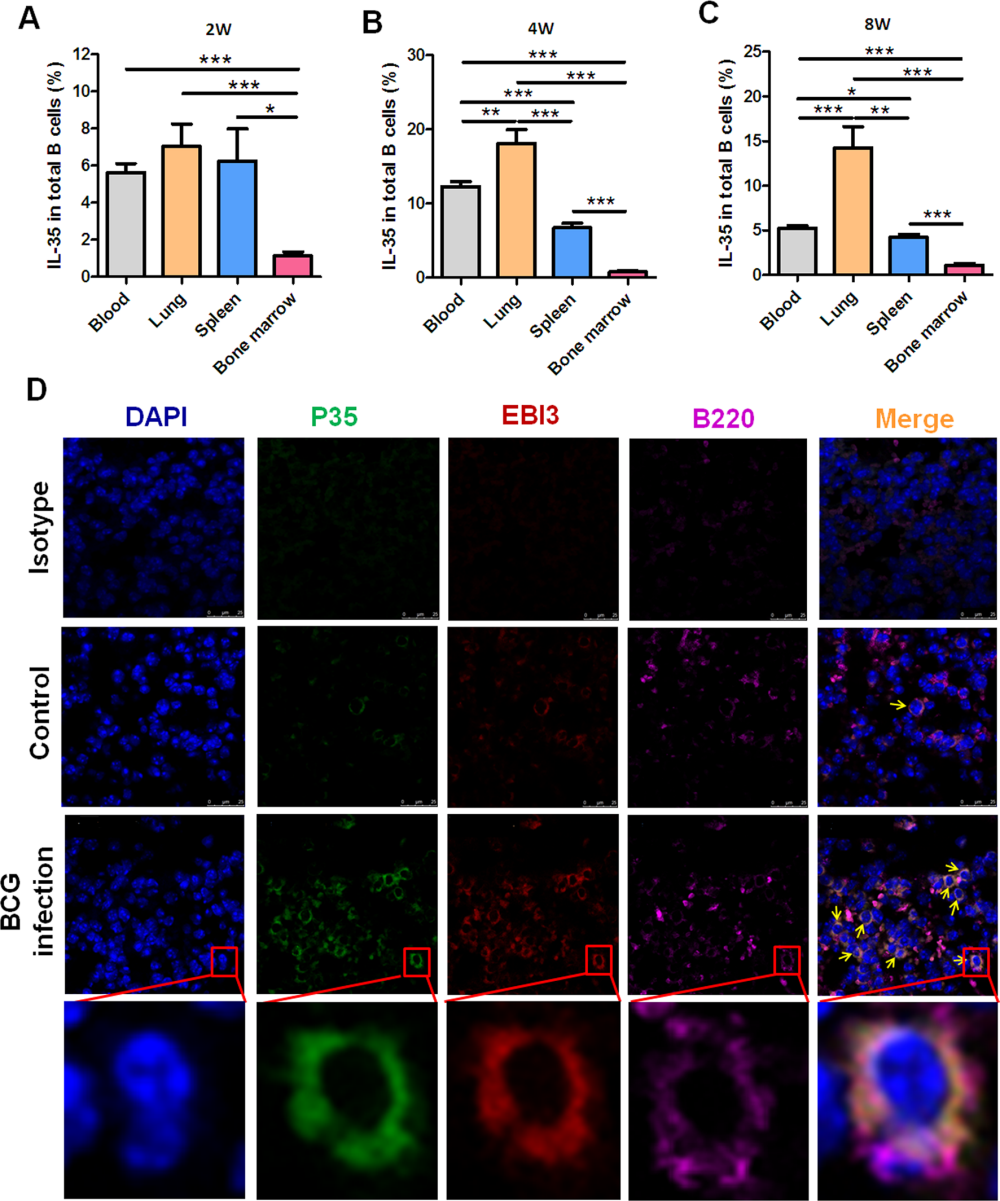
BCG infection promoted the infiltration of IL-35-producing B cells into the lung tissue. The counts of IL-35 producing B cells in the lung tissue were about 1.5 times those in PBMCs after BCG infection for 4 weeks. Further comparative analysis was performed to investigate whether mycobacterial infection could induce the filtration of IL-35-producing B cells into the lung tissue. (**A**–**C**) Bar graph showing the comparison of the frequencies of IL-35-producing B cells in PBMCs (*n* = 10) and mononuclear cells from the lungs (*n* = 6), spleen (*n* = 6), and bone marrow (*n* = 6) after 2 weeks (**A**), 4 weeks (**B**), and 8 weeks (**C**). The *P* value is shown in each column. *P < 0.05; **P < 0.01; ***P < 0.001. Furthermore, confocal IF detection were performed to analyze IL-35-producing B cells in BCG-infected mice or controls with specific antibodies and assessed by laser scanning confocal microscopy (**D**). Co-localization of Ebi3 and p35 was observed by IF staining in B220^+^ B cells. Blue indicates DAPI, green indicates p35, red indicates Ebi3, and pink indicates B220.

The results suggested that the frequency of IL-35-producing B cells in the lung tissue was about 1.5 times higher as that in PBMCs after BCG infection for 4 weeks. Then, whether mycobacterial infection could induce the infiltration of IL-35-producing B cells into lung tissue was analyzed in this study. FCM results showed that the frequencies of p35^+^ and Ebi3^+^ B cells in the lungs were much higher than those in the circulating blood and in the spleen and bone marrow. The frequency of p35^+^Ebi3^+^ B cells reached a peak level 4 weeks after BCG infection. Even after 8 weeks, it remained at a high level (Fig. 3A–C). Furthermore, IF staining with confocal analysis was performed on lung tissue sections of BCG-infected mice to determine the effects of mycobacterial infection on the alterations of IL-35-producing B cells. The surface markers of B cells B220 and two subunit markers of IL-35 (Ebi3 and p35) were stained with different fluorescently labeled antibodies. The results visualized that more Ebi3^+^p35^+^B220^+^ B cells appeared in the lung tissue in BCG-infected mice than in control (Fig. 3D).

Taken together, these results suggested that BCG infection could drive the infiltration of IL-35-producing B cells into the lung tissue in mice.

### Anti-TB therapy reduced the counts of IL-35-producing B cells in BCG-infected mice

**Figure 4:**
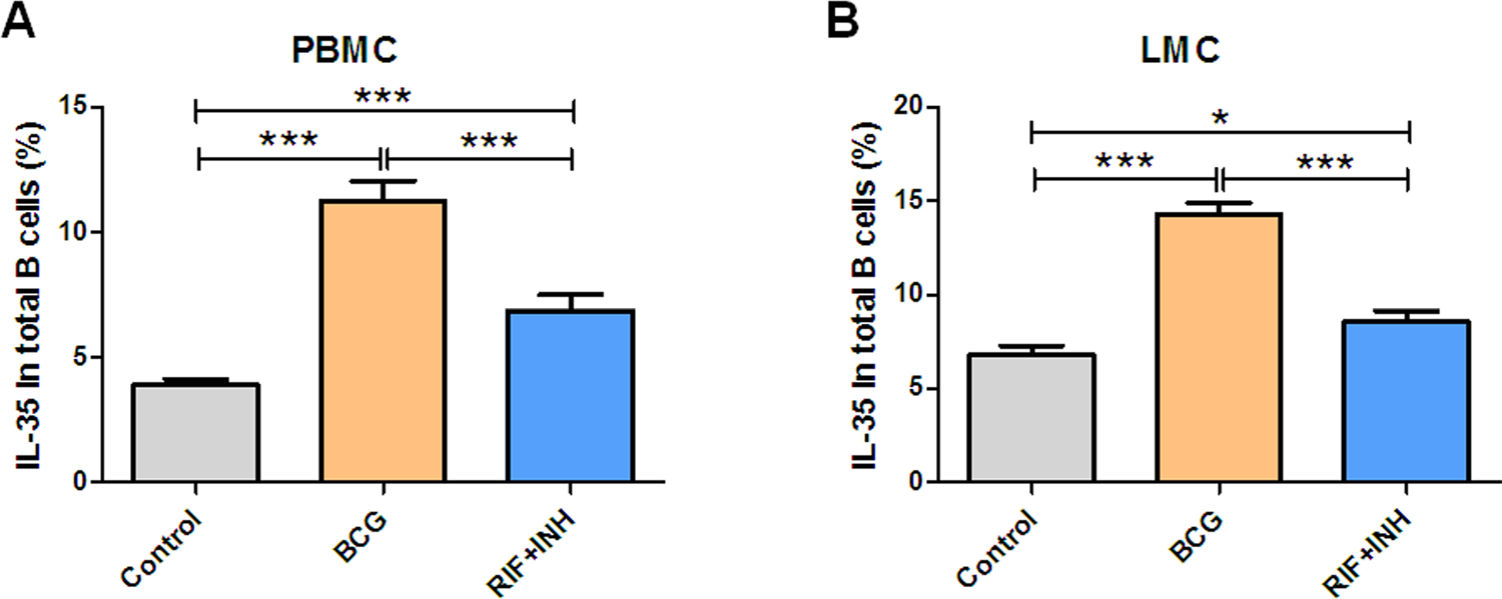
Anti-TB treatment reduced the counts of IL-35-producing B cells in BCG-infected mice. To further confirm the effect of mycobacterial infection on IL-35-producing B cells, the influence of anti-TB therapy on BCG-infected mice was investigated with RIF and INH treatment (*n* = 10). (**A**, **B**) Bar graph showing the comparison of the effects of RIF and INH treatment on the frequencies of IL-35-producing B cells in the blood (**A**) and in the lung tissue (**B**) in BCG-infected mice compared with the controls. The *P* value is shown in each column. *P < 0.05; **P < 0.01; ***P < 0.001.

The present study sought to determine whether treatment with anti-TB drugs could alter IL-35-producing B cells so as to validate the effect of mycobacterial infection on IL-35-producing B cells from the opposite side. Along with the anti-TB drug rifampicin (RIF) and isoniazid (INH) treatment, populations of IL-35-producing B cells reduced not only in PBMCs but also in LMCs compared with mice without anti-TB treatment (Fig. 4). This result further confirmed that mycobacterial infection indeed induced an increase in the counts of IL-35-producing B cells.

### Correlation of the counts of IL-35-producing B cells with bacterial load in the lungs in BCG-infected mice

**Figure 5:**
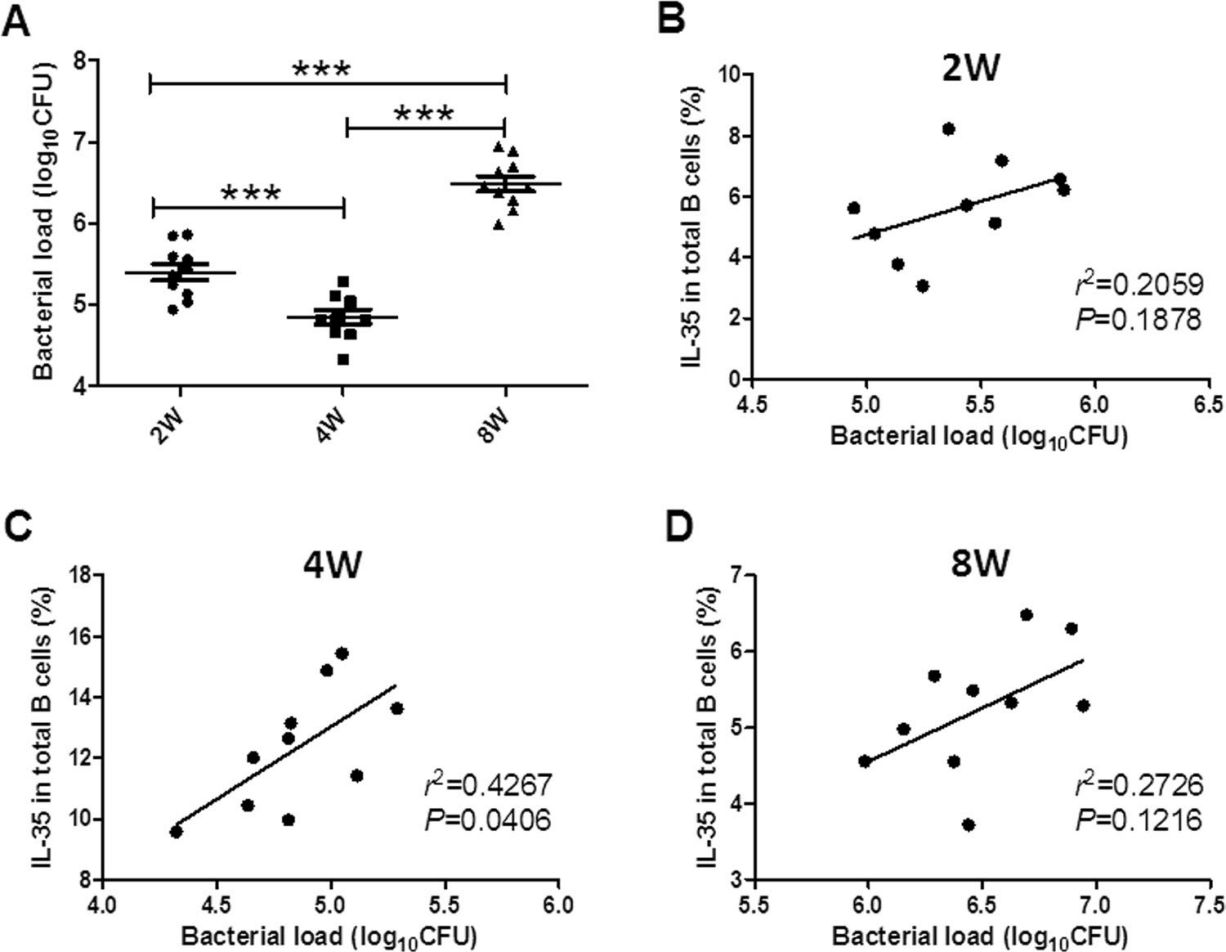
Correlation of the counts of IL-35-producing B cells with bacterial load in the lungs in BCG-infected mice. The correlation of the frequencies of IL-35-producing B cells in PBMCs with the bacterial load in the lungs was analyzed (*n* = 10). (**A**) Showing the bacterial load in the lungs after 2, 4, and 8 weeks. (**B**–**D**) Showing correlation of the counts of circulating IL-35-producing B cells with the bacterial load 2 weeks (**B**), 4 weeks (**C**), and 8 weeks (**D**). The *P* value is shown in each column, *P < 0.05; **P < 0.01; and ***P < 0.001, or in each graph directly.

Previous reports indicated that IL-35-producing B cells possessed negative immune regulatory effects, for example, the downregulation of the development of autoimmune diseases^27^. However, the relationship between IL-35-producing B cells and the bacterium-clearing function in the lungs in pulmonary tuberculosis has not been reported yet. The mycobacterial colony formation unit count method was used in this study to calculate the bacilli number in the lung tissue from BCG-infected mice to determine whether IL-35-producing B cells affected the bactericidal function in vivo. The bacterial numbers were different 2, 4, and 8 weeks after bacterial injection; it was lowest after 4 weeks (Fig. 5A). The percentage of IL-35-producing B cells in the blood positively correlated with bacterial load 4 weeks after BCG injection Fig. 5B–D). These results suggested that IL-35-producing B cells might downregulate the bactericidal ability in BCG-infected mice.

### BCG infection induced IL-10 production in IL-35-producing B cells

**Figure 6:**
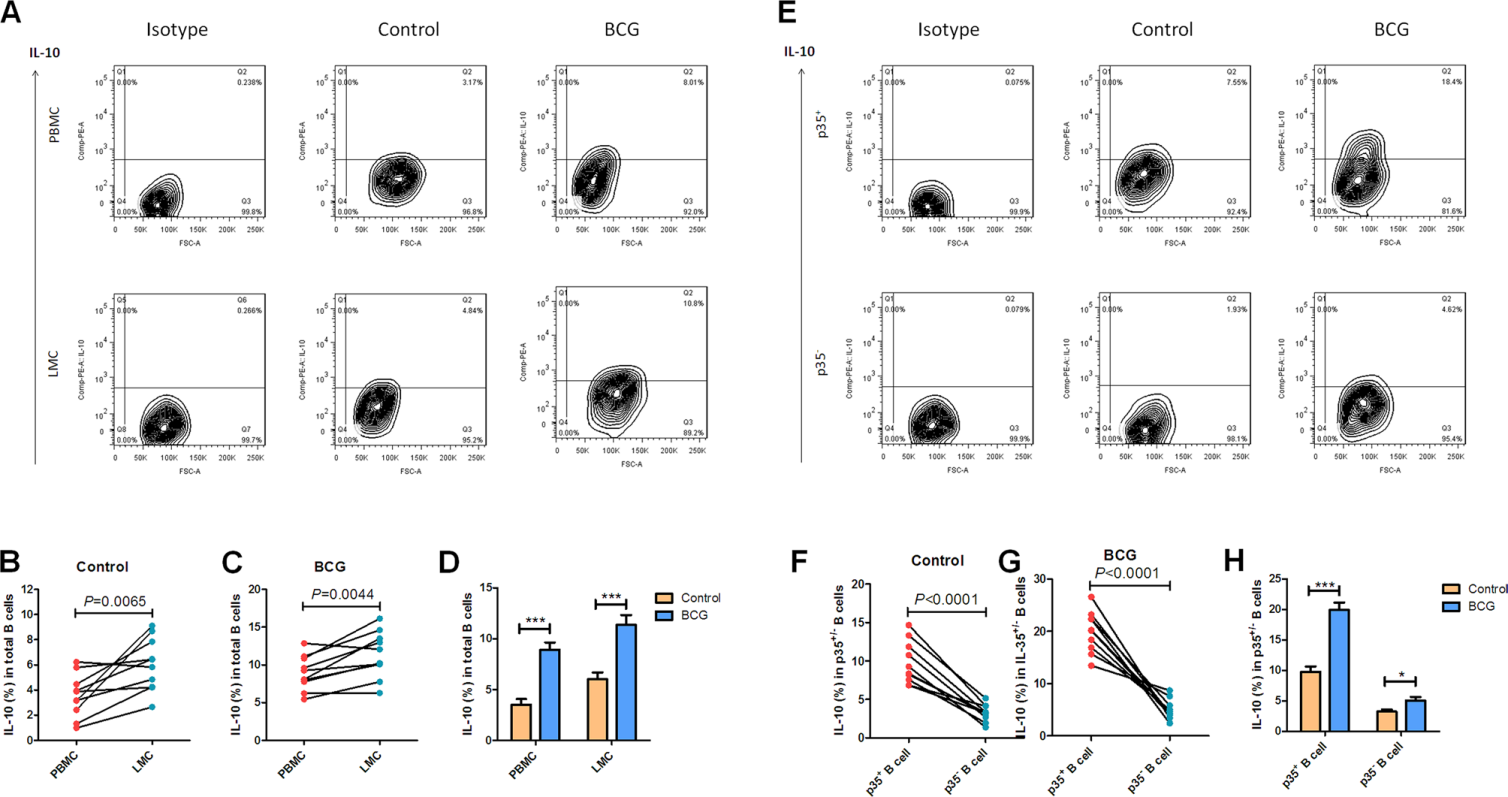
BCG infection increased IL-10 production in IL-35-producing B cells. The production of IL-10 in B cells was determined by intracellular cytokine flow cytometry. PBMCs or LMC was extracted from BCG-infected mice (n = 10). Then, the frequencies of IL-10-producing in total B cells or in CD19^+^ p35^+^_B cells were analyzed by flow cytometry. The gating strategy is shown in Figure S3 (*n* = 10). (**A**, **E**) shows the representative histograms for flow cytometry analysis of the expression of IL-10 in B cells in PBMCs and B cells in LMCs (**A**), and the expression of IL-10 in p35 expressed B cells or in p35 nagative B cells in PBMCs (**E**) in BCG-infected mice and controls for 4 weeks. (**B**, **C**) Paired comparison of the frequency of IL-10-producing CD19^+^ B cells between blood and lung in controls (**B**) and BCG-infected mice (**C**). (**D**) Graph data showing the comparison of the frequency of IL-10-producing CD19^+^ B cells between BCG-infected mice and controls. (**F**, **G**) Paired comparison of the frequency of IL-10-positive cells between p35-expressing B cells and (**F**) p35 negative B cells (**G**) in PBMCs. (**H**) Graph data showing the comparison of the frequency of IL-10-producing cells in p35-expressing or in p35 negative B cells between BCG-infected mice and controls. The *P* value is shown in each column, *P < 0.05; **P < 0.01; and ***P < 0.001, or in each graph directly.

IL-35 negatively regulated the development of autoimmune disease by inducing the generation and expansion of IL-10-producing B cells^26^. A previous study indicated that IL-35-producing B cells in patients with active TB possessed a stronger ability to produce more IL-10^33^. This study also aimed to determine whether the mycobacterial infection in mice could induce IL-10 production in IL-35-producing B cells. Direct ICS with flow cytometry was used to test the frequencies of IL-10-positive B cells in PBMCs and LMCs from BCG-infected mice. The results showed that the frequency of IL-10-positive B cells in LMCs were higher than those in PBMCs both in BCG-infected mice and in controls (Fig. 6A,B). The frequencies of IL-10-positive B cells in BCG-infected mice, in both the blood and lung tissue, were much higher than that in controls (Fig. 6C). Notably, IL-35-producing B cells in the blood, not only in controls but also in BCG-infected mice, possessed strong ability to produce IL-10, displaying that the frequencies of IL-10-positive B cells in IL-35-producing B cells were much higher than that in IL-35-negative B cells both in BCG-infected mice and in controls (Fig. 6D,E). Importantly, BCG infection induced more IL-10 production in IL-35-producing B cells than in controls (Fig. 6F). These results indicated that BCG infection induced a high level of IL-10 production in IL-35-producing B cells.

### Exogenous IL-35-induced elevation in the frequencies of IL-35-producing B cells and their infiltration into the lungs were associated with the downregulation of Th1/Th17 and upregulation of Foxp3^+^ Treg

**Figure 7:**
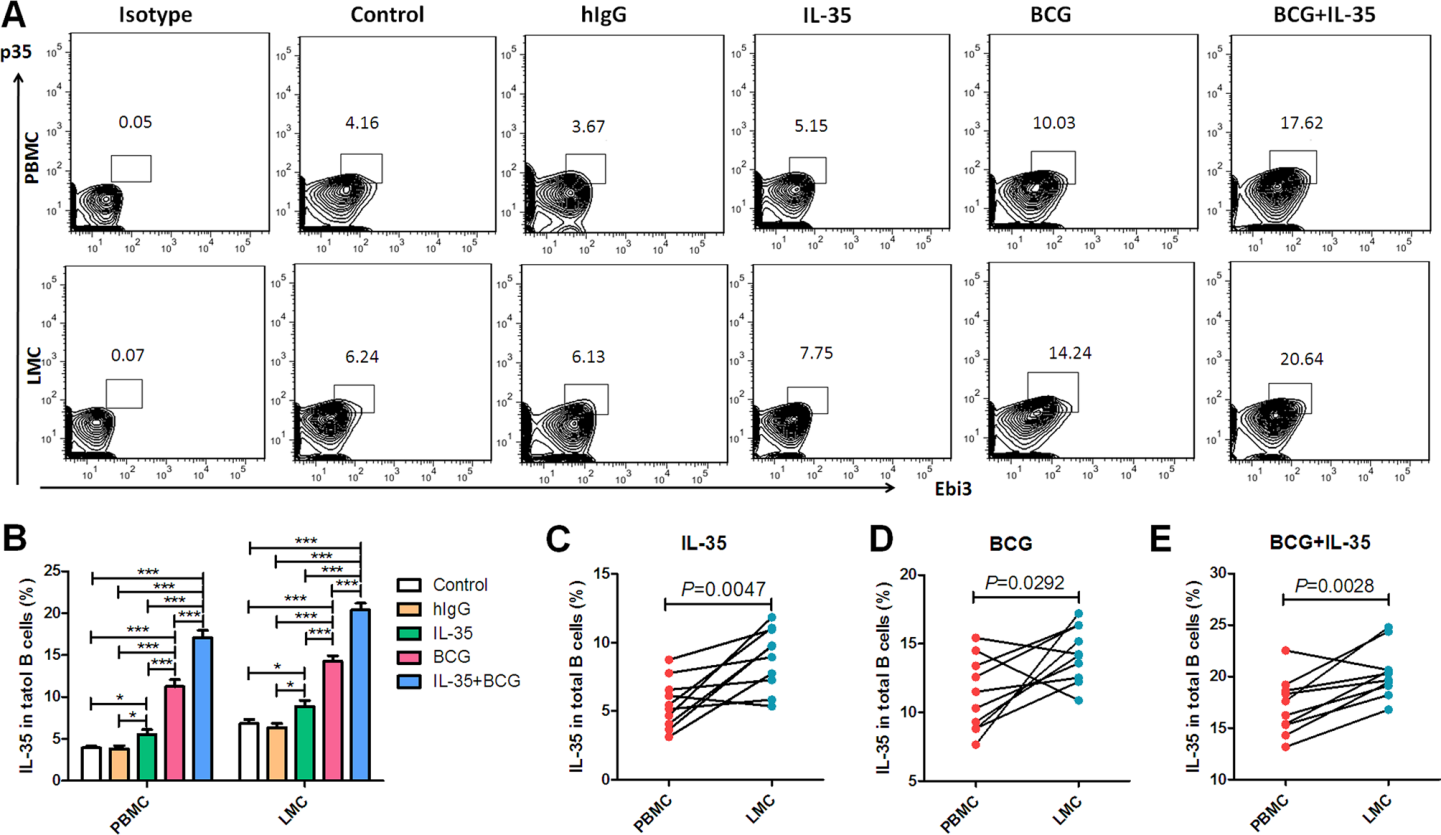
Exogenous IL-35 driven elevation in the counts of IL-35-producing B cells and their infiltration into the lungs. IL-35-producing B cells possess autocrine or paracrine characteristics to induce the proliferation of IL-35-producing B cells. This study sought to test whether exogenous IL-35 could induce an elevation in the counts of IL-35-producing B cells (*n* = 10). (**A**) Representative histograms for flow cytometry analysis of IL-35-producing B cells in the circulating blood (PBMC) and in the lung tissue (LMC) 4 weeks after BCG infection and exogenous IL-35 treatment. (**B**) Showing effect of exogenous IL-35 on the frequencies of IL-35-producing B cells in the circulating blood and lung tissue. (**C**–**E**) Showing paired comparison of IL-35-producing B cells between the blood and lungs after treatment with IL-35 (**C**), BCG (**D**), and BCG + IL-35 (E). The *P* value is shown in each column,*P < 0.05, **P < 0.01; and ***P < 0.001, or in each graph directly.

**Figure 8:**
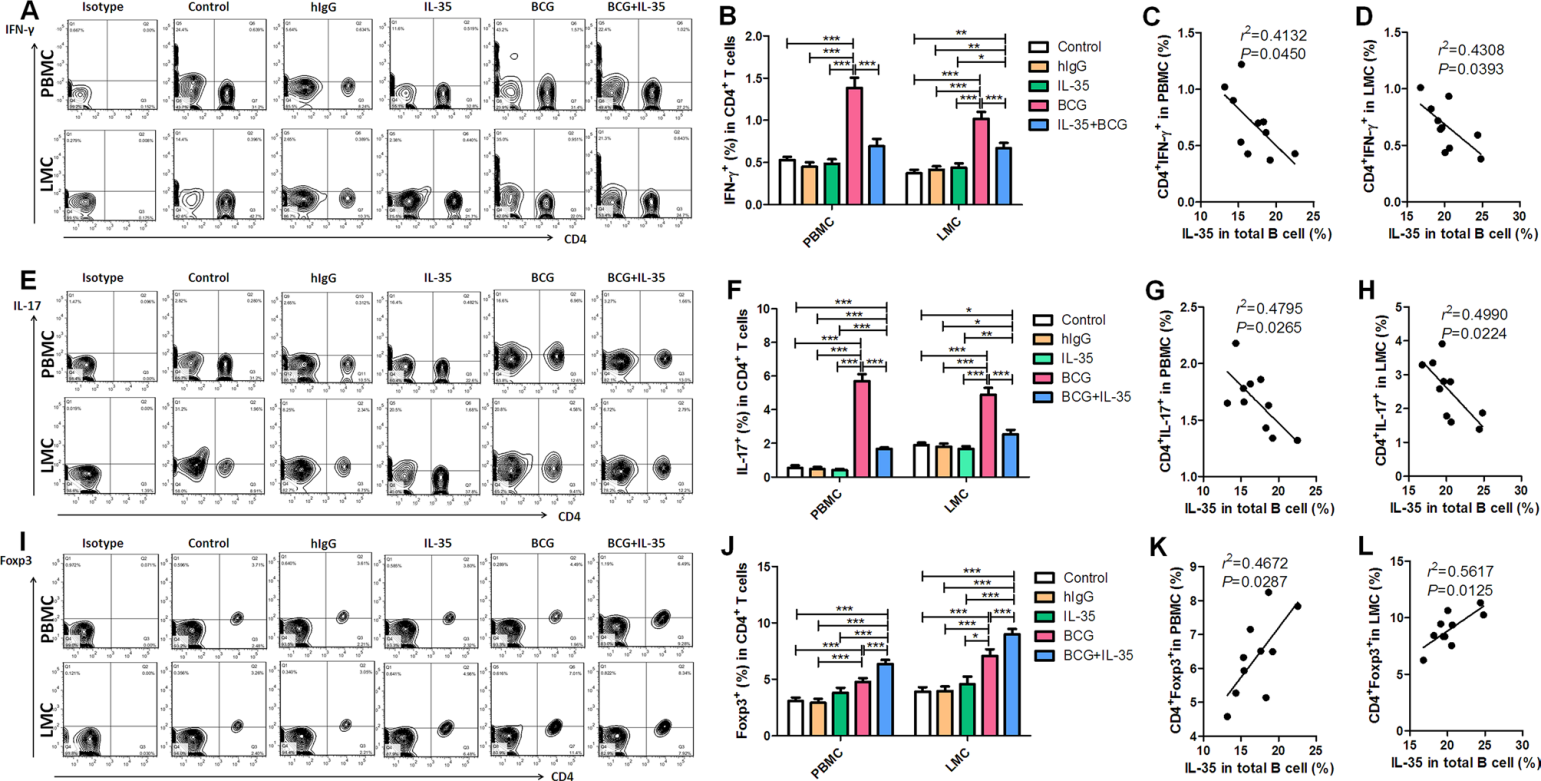
Exogenous IL-35-driven elevation in the counts of IL-35-producing B cells was associated with the downregulation of Th1/Th17 cells and upregulation of Foxp3^+^ regulatory T cells. Earlier studies reported that IL-35-producing B cells had a strong negative immune regulatory ability to affect T cells and induce the proliferation of regulatory T cells (Treg). This study sought to analyze whether the exogenous IL-35 could affect effector Th1/Th17 and Treg and the relationship between the elevation of the counts of IL-35-producing B cells and the frequencies of effector Th1/Th17 and Treg in the peripheral blood and lung tissue (*n* = 10). (A, E, and I) Representative histogram for the flow cytometry analysis of Th1 (IFN-γ stain) (**A**), Th17 (IL-17 stain) (**E**), and Treg (Foxp3 stain) (**I**) in the circulating blood and lung tissue 4 weeks after BCG infection and exogenous IL-35 treatment, as detected by ICS. (**B**, **F**, **J**) Showing the effects of exogenous IL-35 on the frequencies of Th1 (**B**), Th17 (**F**), and Treg (**J**) in the circulating blood and lung tissue. (**C**, **G**, **K**) Correlation of IL-35-producing B cells in PBMCs to Th1 (**C**), (Th17) (**G**), and Treg (**K**) in PBMCs, respectively. (**D**, **H**, **L**) Correlation of IL-35-producing B cells in LMCs with Th1 (D), (Th17) (**H**), and Treg (**L**) in LMCs, respectively. The *P* value is shown in each column, *P < 0.05, **P < 0.01; and ***P < 0.001, or in each graph directly.

BCG infection could induce an elevation in the frequencies of IL-35-producing B cells in the blood and lung tissue, which correlated with bactericidal ability in *Mycobacterium*-infected mice. Then, the effects of IL-35-producing B cells on bactericidal ability were explored. IL-35 is the dominant effector cytokine of IL-35-producing cells with autocrine or paracrine ability to induce the increased secretion of IL-35 and IL-10 by B cells, negatively downregulate effector T cell function, and induce Treg proliferation^26,27,32^. Exogenous IL-35 injection was given to BCG-infected mice for examining the impacts of IL-35 on IL-35-producing B cells and further analyzing its effect on Th1/Th17 and Treg. The results showed that exogenous IL-35 treatment increased the percentages of IL-35-producing B cells in PBMCs and LMCs in BCG-infected mice, which were much higher than those in BCG-infected mice without exogenous IL-35 treatment (Fig. 7A,B). Consistent with the previous results, paired comparison analysis further confirmed that the percentage of IL-35-producing B cells was higher in the lung tissue than in the circulating blood (Fig. 7C–E), and exogenous IL-35 treatment promoted the infiltration of IL-35-producing B cells into the lung tissue (Fig. 7E). Exogenous IL-35 treatment obviously impacted Th1/Th17, despite BCG infection–induced elevation in the percentages of anti-tuberculosis effector cells Th1/Th17, displaying that the frequencies of IFN-γ/IL-17-positive cells in CD4 + T cells increased in BCG-infected mice compared with controls (Fig. 8A,B and E,F, respectively). These findings implied that the frequencies of IFN-γ- or IL-17-positive cells in CD4^+^ T cells were much lower in BCG + IL-35-treated group than in the BCG only-treated group (Fig. 8A,B and E,F, respectively). Interestingly, exogenous IL-35 treatment obviously induced elevation in the frequency of Treg cells in the peripheral blood and lung tissue, indicating that the frequency of Treg cells was much higher in the BCG + IL-35-treated group than in the BCG only-treated group (Fig. 8I,J). In addition, comparison of exogenous IL-35 treatment—driven Treg infiltration in the lung tissue showed that the frequency of Treg cells in the lung tissue was much higher than that in the blood (Fig. 8I,J). Furthermore, exogenous IL-35 treatment likely impacted Th1/Th17 and Treg via inducing the elevation in the counts of IL-35-producing B cells because the frequency of IL-35-producing B cells negatively correlated with the percentage of IFN-γ-positive or IL-17-positive cells in CD4^+^ T cells (Fig. 8C, D and G, H, respectively) and positively correlated with Foxp3^+^ Treg (Fig. 8K,L).

Thus, these data suggested that IL-35 could trigger the elevation in the counts of IL-35-producing B cells, promote their infiltration into lung tissue, and downregulate anti-tuberculosis effector cell Th1/Th17 infiltration. Further, it drove Foxp3^+^ Treg elevation and infiltration into lung tissue.

## Discussion

This study was performed based on a recent report about the patients with active TB who had IL-35-producing B cells with a stronger ability to secrete IL-10. The findings were as follows: (1) *M. bovis* BCG infection elevated the percentages of IL-35-producing B cells, a novel subset of regulatory B cells, and promoted their infiltration into the lung tissue. Also, anti-TB chemotherapy control of BCG infections could reverse the counts of IL-35-producing B cells in the peripheral blood and lung tissue. Elevated counts of IL-35-producing B cells negatively correlated with the bactericidal ability in mycobacterial infection. (2) Exogenous IL-35 treatment could induce the elevation in the counts of IL-35-producing B cells and promote their infiltration into the lung tissue, further downregulating the infiltration of anti-tuberculosis effector cell Th1/Th17 and upregulating the infiltration of Foxp3^+^ regulatory T cells into the lung tissue.

More recently, the counts of IL-35-producing B cells were found to increase in the peripheral blood and tuberculosis granuloma in patients with active TB^33^. IL-35^+^CD20^+^B cells were found within the tuberculous granuloma of ATB patients by Immunohistochemical (IHC) and immunofluorescense (IF) staining and the higher levels of both subunits of IL-35 (p35 and Ebi3) in purified B cells from ATB patients were also found in mRNA analysis. In vitro experiments further demonstrated that purified B cells from ATB patients generated more IL-35 after stimulated by *M. tuberculosis* (M.tb) lysis. Moreover, flow cytometer analysis displayed that the IL-35-producing B cells from ATB patients secreted more IL-10. The present study further provided intriguing data illustrating that the mRNA expression of IL-35 subsets *p35* and *Ebi3* was elevated in the peripheral blood and predominant immune organs. Notably, it also increased in the target organ of infection by *Mycobacterium* spp., the lungs. Consistently, the results of ICS also showed that the frequencies of p35- and Ebi3-co-expressing B cells increased in the peripheral blood and lungs. Despite the lack of reports on IL-35-producing B cells in infectious diseases, mice in which only B cells did not express IL-35 displayed a strikingly improved resistance to infection with the intracellular bacterial pathogen *Salmonella typhimurium*, as shown by their superior containment of the bacterial growth and their prolonged survival both after primary infection, and upon secondary challenge after vaccination, compared to control mice. During Salmonella infection IL-35- and IL-10-producing B cells corresponded to plasma cells expressing the transcription factor Blimp1^26^. Moreover, the counts of IL-35-expressing B cells were found to be elevated in patients with *M.leprae* infection, another kind of mycobacterial infectious diseases^41^. Intriguingly, the present study also found that the frequency of IL-35-producing B cells reduced in PBMCs and in the lung tissue with anti-TB chemotherapy using RIF and INH. Thus, this study further confirmed a recent finding in a BCG-infected animal model that mycobacterial infection could drive the elevation in the frequency of IL-35-producing B cells in the peripheral blood and promote their infiltration into the lung tissue. The control of bacilli infection using anti-TB chemotherapy could downregulate IL-35-producing B cells. The elevation of the counts of IL-35-producing B cells suggested that it should affect the function of host defense against mycobacterial infection. Early studies supported that the elevation in the counts of IL-35-expressing B cells positively correlated with the bacteriological index in patients with *M.leprae*^41^. Also, mice in which only B cells did not express IL-35 displayed a markedly improved resistance to infection with the intracellular bacterial pathogen *Salmonella enteric*compared with control mice^26^.The present study demonstrated that the frequency of IL-35-producing B cells positively correlated with the bacterial load in the lung tissue, indicating that the elevation in the counts of IL-35-producing B cells was associated with the downregulation of bactericidal activity in mycobacterial infection in vivo.

In view of the negative immune regulatory function of IL-35, various studies focused on the underlying mechanism related to IL-35. IL-35 has the ability to suppress T cell proliferation and functions as well as Th17 responses^42^. It can also induce the expansion of IL-10-/IL-35-expressing T and B cells, thereby causing further suppression effect within the immune system^27,43^. Furthermore, not only the heterodimer of IL-35 but its subunit p35 or Ebi3 possesses the anti-inflammatory capacity^44–46^. Recombinant IL-35 can stimulate Breg cells to secrete IL-10 and IL-35^26,27^. Interestingly, Recombinant IL-35 stimulation can dampene cytotoxicity and interferon-γ production in both direct and indirect contact co-culture systems in viral hepatitis-induced acute-on-chronic liver failure^47^. For further understanding the role of IL-35-producing B cells in mycobacterial infection, exogenous recombinant IL-35 was used in this study to treat BCG-infected mice, and its effects were validated. Treatment with exogenous IL-35 could induce more IL-35-producing B cells in the peripheral blood and lung tissue. Moreover, about 22% of B cells co-expressing p35 and Ebi3 existed in the lung tissue in BCG-infected mice treated with IL-35, which was much higher than that in mice without IL-35 treatment (about 14%). This suggested that exogenous IL-35 treatment not only caused the elevation in the counts of IL-35-producing B cells but also promoted their infiltration into the lung tissue. These results were also consistent with the recent finding of the prominent infiltration of IL-35-producing B cells into the TB granuloma of patients with ATB. These results might confirm, to some extent, that IL-35-producing B cells possessed autocrine or paracrine characteristics to induce the proliferation of IL-35-producing B cells.

Then, experiments were conducted to analyze the relationship between IL-35-producing B cells and T cells. Concomitant with the elevation in the counts of IL-35-producing B cells in the peripheral blood and lung tissue, the frequencies of IFN-γ-producing CD4^+^ T cells (Th1) and IL-17-producing CD4^+^ T cells (Th17) decreased. Reciprocally, the frequency of Foxp3^+^ Treg cells increased in the peripheral blood and lung tissue. In addition, the frequency of IL-35-producing B cells displayed a negative correlation withTh1/Th17, but a positive correlation with Treg.

The present study showed that the frequencies of IL-10-positive B cells in BCG-infected mice were much higher than those in controls and the frequencies of IL-10-positive B cells in IL-35-producing B cells were much higher than those in IL-35-negative B cells both in BCG-infected mice and in controls (Fig. 6D,E). Of note, BCG infection caused more IL-10 production in IL-35-producing B cells than in controls, displaying that the frequency of IL-10-positive B cells in IL-35-producing B cells was much higher than that in controls (Fig. 6F). IL-10-producing Breg cells constitute a major population of B cells that is associated with the development of autoimmune diseases and cancers^17–21^. A previous study also found that IL-35 negatively regulated the development of autoimmune diseases by inducing the generation and expansion of IL-10-producing B cells^27^. During *Salmonella* infection and EAE, IL-35- and IL-10-producing B cells were the main source of B-cell-derived IL-35 and IL-10^26^. Prominent IL-10^+^ B cells have been found in patients with active TB^48^^,^ and *M. tuberculosis* mannose-capped lipoarabinomannan induces IL-10-producing B cells and hinders CD4^+^ Th1 immunity^25^. A previous study demonstrated that IL-35-producing B cells from patients with active TB possessed a stronger ability to produce IL-10^33^. Thus, the data further suggest that BCG infection induced high levels of IL-10 production in IL-35-producing B cells which might be the mechanism underlying the downregulation of immunity against mycobacterial infection.

The present study expanded previously undescribed findings in a *Mycobacterium-*infected mouse model to confirm the results of a recent study on patients with active TB showing the elevation in the counts of IL-35-producing B cells in the blood and their infiltration into TB lesion tissue. The findings also extended the observations that elevated counts of IL-35-producing B cells downregulated the bactericidal activity in mycobacterial infection in vivo. The underlying mechanisms might be associated with the autocrine or paracrine characteristic of IL-35-producing B cells to secrete high levels of IL-35 and IL-10, and also their tendency to inhibit the function of effector Th1/Th17 cells and promote the function of regulatory Foxp3^+^ Treg cells. However, the underlying mechanisms discussed in the present study were deduced from the association between IL-35-producing B cells and effector/regulatory anti-tuberculosis T cells. Further studies should be performed with knockout mice or using siRNA assay to silence the expression of IL-35, so as to clarify whether the absence of IL-35-producing B cells could be important to TB progression.

## Supplementary information

Supplementary Legends.

Supplementary Figure S1.

Supplementary Figure S2.

Supplementary Figure S3.
