# Supplementary material for: Elevation in the counts of IL-35-producing B cells infiltrating into lung tissue in mycobacterial infection is associated with the downregulation of Th1/Th17 and upregulation of Foxp3+Treg
Source: Sci Rep. 2020 Aug 6;10:13212. doi: 10.1038/s41598-020-69984-y (PMC7411070; doi:10.1038/s41598-020-69984-y)
Supplement: Supplementary file 1 — Supplementary Legends. [file 41598_2020_69984_MOESM1_ESM.docx]

**Figure S1. General characteristics of BCG-infected mice.**

(A) Size comparison of the spleen between BCG-infected mice and controls. (B) Bodyweight comparison between BCG-infected mice and controls. (C) Spleen weight factor comparison between BCG-infected mice and controls. (D) Histological comparison in the lung tissue between BCG-infected mice and controls (×200, HE stain). (E) Lung tissue of BCG-infected mice stained by acid-fast staining, with red arrow indicating acid-fast bacillus. The Figures were drawn by using Microsoft Office Powerpoint 2007.

**Figure S2. Flow cytometry gating strategy for analysis of IL-35-producing B cells.**

PBMCs or mononuclear cells from the lung, spleen, and bone marrow were gated on FSC and SSC dot plots for lymphocytes. Then, B220-Percp and CD19-PE were gated on B cells by B220 and CD19 expression. Moreover, Ebi3 (APC) and p35 (FITC) expression was analyzed in B cells by ICS.

**Figure S3. Flow cytometry gating strategy for analysis of IL-10-producing B cells.**

PBMCs or mononuclear cells from the lung were gated on FSC and SSC dot plots for lymphocytes. Then, CD19-FITC was gated on B cells by CD19 expression, Then, p35-APC was gated in B cells by p35 expression analyzed in B cells by ICS as p35^+^ B cells. Finally, IL-10 expression was analyzed in total B cells or in p35^+^ B cells by ICS.
