# Supplementary figures and images for: Elevation in the counts of IL-35-producing B cells infiltrating into lung tissue in mycobacterial infection is associated with the downregulation of Th1/Th17 and upregulation of Foxp3+Treg
Source: Sci Rep. 2020 Aug 6;10:13212. doi: 10.1038/s41598-020-69984-y (PMC7411070; doi:10.1038/s41598-020-69984-y)

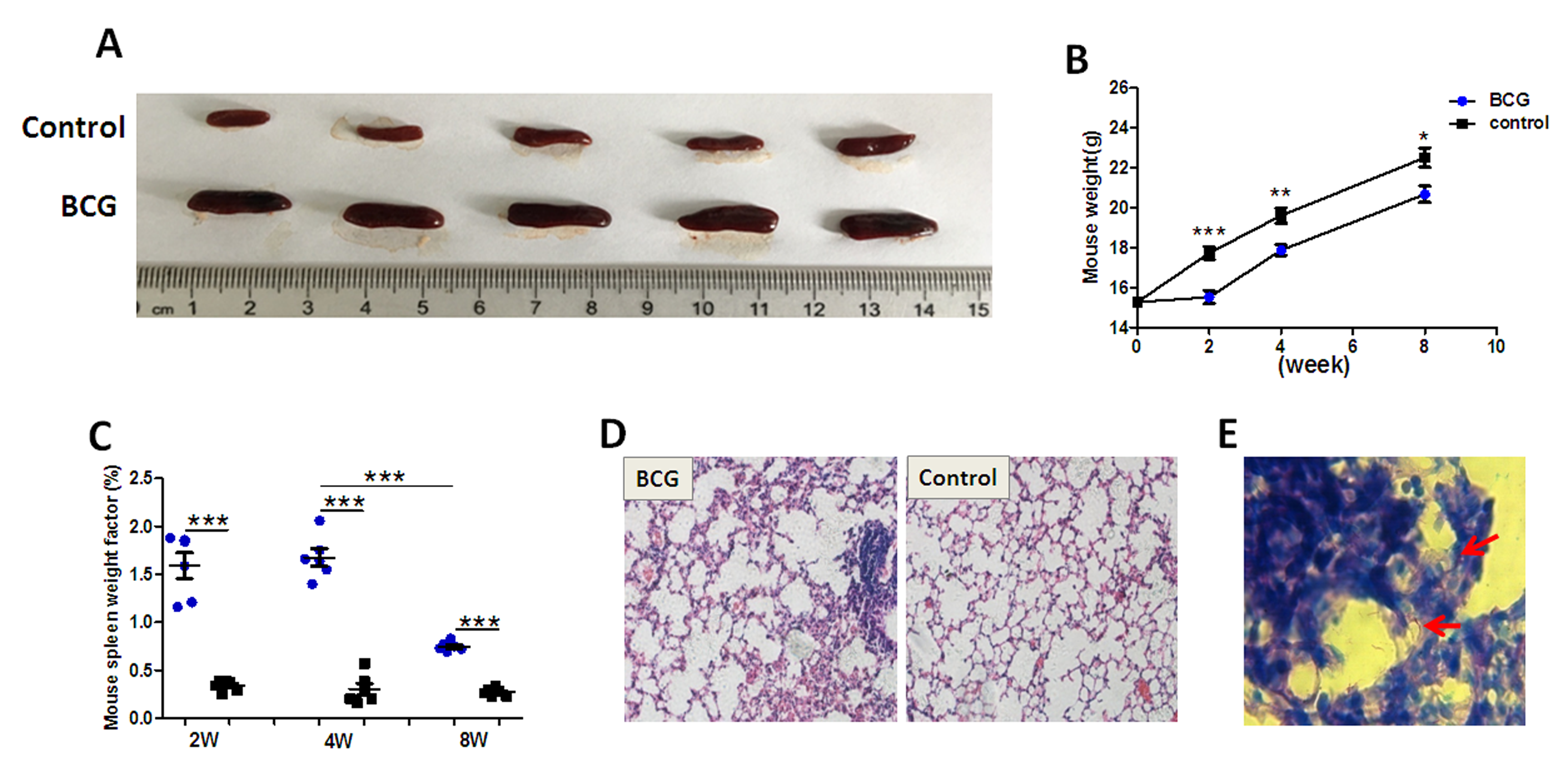

Supplement: Supplementary file 2 — Supplementary Figure S1. [file 41598_2020_69984_MOESM2_ESM.tif]

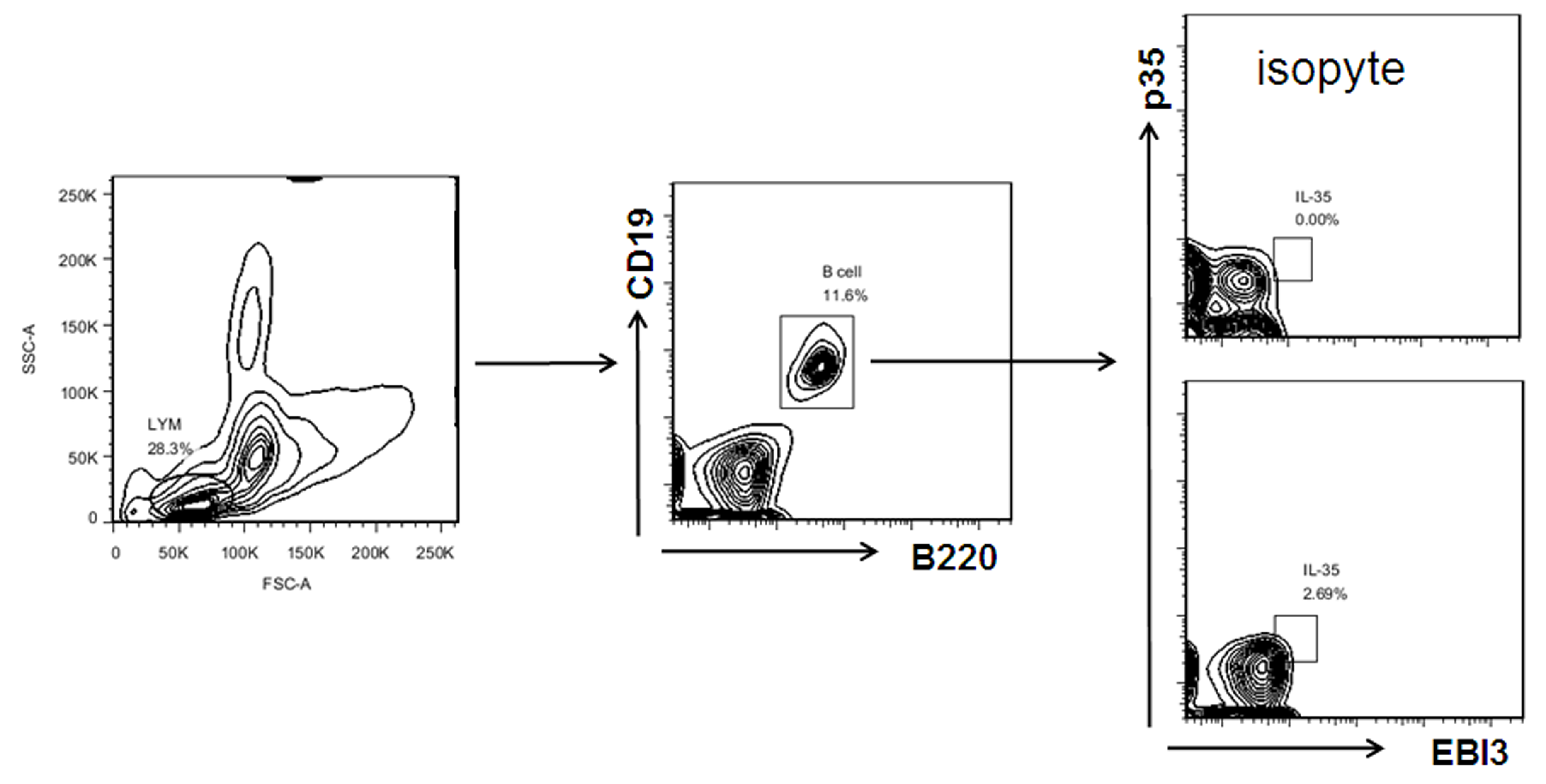

Supplement: Supplementary file 3 — Supplementary Figure S2. [file 41598_2020_69984_MOESM3_ESM.tif]

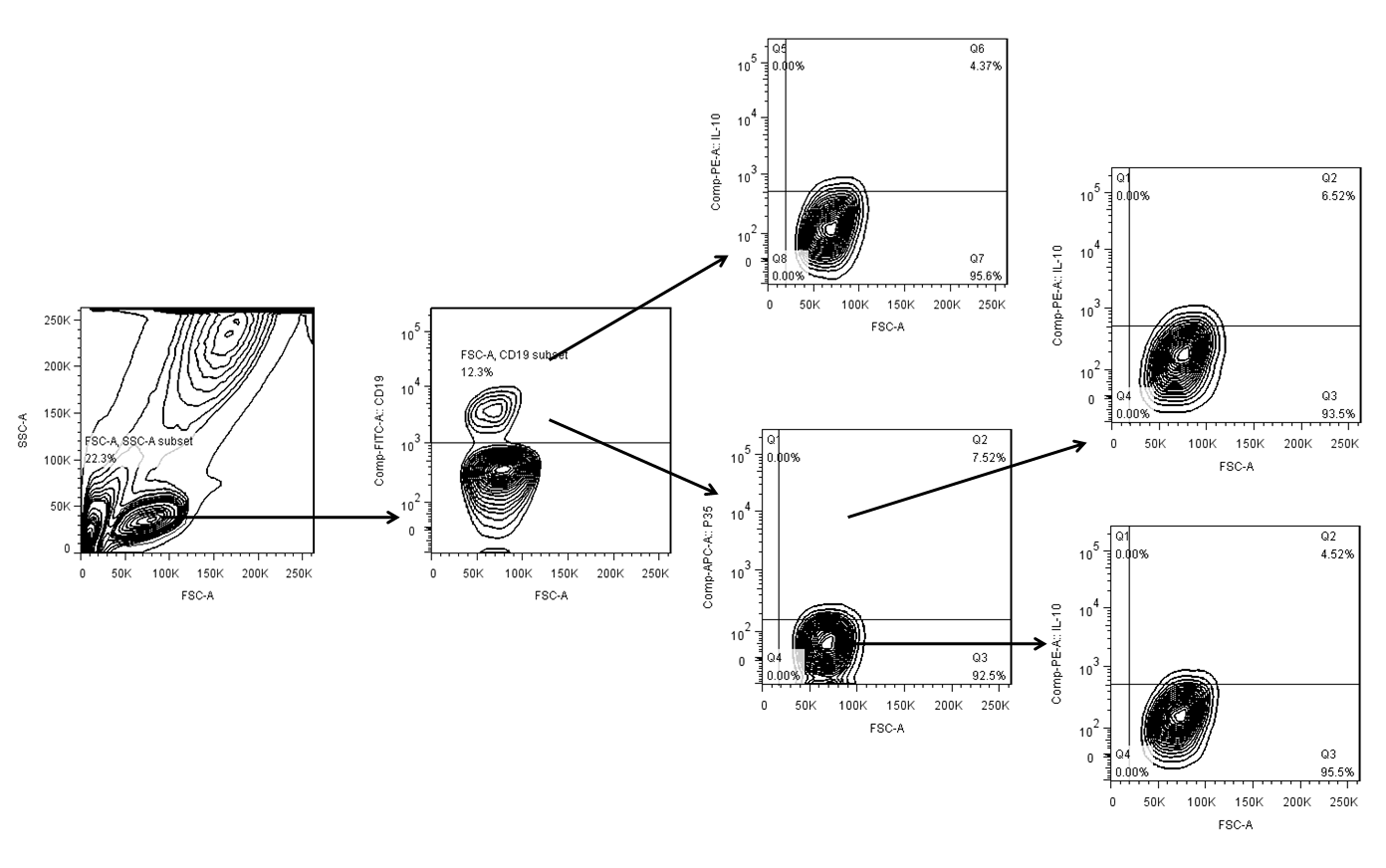

Supplement: Supplementary file 4 — Supplementary Figure S3. [file 41598_2020_69984_MOESM4_ESM.tif]
